# Manufacturing and compatibilization of binary blends of superheated steam treated jute and poly (lactic acid) biocomposites by melt-blending technique

**DOI:** 10.1016/j.heliyon.2022.e09923

**Published:** 2022-07-13

**Authors:** Md. Abdul Alim, Md. Moniruzzaman, Md. Muzaher Hossain, Wahiduzzaman, Md. Reazuddin Repon, Ismail Hossain, Mohammad Abdul Jalil

**Affiliations:** a Department of Textile Engineering, Khulna University of Engineering & Technology, Khulna 9203, Bangladesh; b ZR Research Institute for Advanced Materials, Sherpur 2100, Bangladesh; c Department of Textile Engineering, Khwaja Yunus Ali University, Sirajganj 6751, Bangladesh; d Department of Production Engineering, Faculty of Mechanical Engineering and Design, Kaunas University of Technology, Studentų 56, LT-51424 Kaunas, Lithuania

**Keywords:** Biocomposite, Jute fiber, Poly (lactic acid), Superheated steam, Swelling

## Abstract

This work investigated the effect of superheated steam (SHS) jute fiber and poly (lactic acid) (PLA) having a weight proportion of 30:70 which were synthesized using the melt blending method. The goal of this treatment was to boost up the fiber-polymer interfacial linkage. The action was conducted in a superheated steam oven at various times (30–120 min) and temperatures (170–220 °C). The biocomposites were assessed in terms of mechanical characteristics, dimensional stability and morphological properties. Compared to different treatment temperatures, the results showed that treatment at 210 °C for 60 min offered the best tensile characteristics. Because of the presence of SHS-Jute, the tensile, impact, bending and dimensional stability of the bio-composites have been improved. The FTIR and SEM study revealed progress in the interfacial linkage between SHS-Jute and PLA. This interfacial link improves the bending strength of SHS-Jute-PLA biocomposites by about 15.64%. X-ray diffraction (XRD) investigation also showed an elevation in the crystalline structure with the incorporation of SHS-Jute. The degradation tests of the biocomposite were carried out in deionized water. SHS treatment reduces hemicellulose contents in jute fiber which causes water uptake% reduction is 54% in SHS-Jute-PLA. The SHS-Jute-PLA biocomposite appeared with promising characteristics for utilization as a green and ecological substitute particle board material.

## 1 Introduction

The world is approaching green and sustainable developments and arousing interest in high-performance, environmentally friendly, and biodegradable materials. For this, natural fibers attract the attention of both academics and industry [1, 2]. Although synthetic fibers provide high mechanical performance, they are unwelcomed due to environmental issues. Natural fibers supplant synthetic fibers as natural fibers offer the excellent properties including light weight, renewable resource, reasonable strength, superabundant, low density, non-irritating to skin and eye, inexpensive, bio-degradable, stiffness, non-toxic and eco-friendly [3, 4, 5, 6]. The demand for light-weight natural fiber composites is increasing rapidly, especially in the automotive industry, which attributes weight reduction and therefore low fuel consumption [7].

Among all other lignocellulosic fibers, jute is more promising in the field of composites or biocomposites due to its accessibility, specified mechanical properties and obviously low cost [8, 9]. Jute fiber has (58–63%) cellulose, (12–15%) lignin, (20–24%) hemicellulose and a small amount of pectins, waxes and fats [9, 10, 11, 12, 13]. For a higher cellulose content, the stiffness property of jute is higher, which helps to improve deflection under extreme load and transfer stress to the fibers [11]. In addition, jute fiber exhibits numerous properties including non-abrasive, low density, least processing damage, biodegradable, greater aspect ratio, combustible, and insulation [8, 9, 11, 14]. Jute fiber also faces some challenges, such as poor strength, having a strong preference for water absorption, thermal degradation, etc. Composites made from jute experience lower strength and poorer bonding with the matrix materials. Various physical and chemical surface treatments were introduced to enhance the properties of the composite by ensuring a strong interfacial bond between the fiber and the matrix [15]. Bangladesh, Burma, India, Vietnam, China, Uzbekistan, and Nepal mainly produce jute fiber. According to FAO 2020, India (1,807,264 tonnes) is the highest volume of jute producing country, Bangladesh (804,520 tonnes) is in the 2nd position, and Uzbekistan (19,122 tonnes) is in the 3rd position. And global production is about more than 3.3 million tons per year. Jute contains hydroxyl groups in cellulose that make jute hydrophilic.

A complete biodegradable composite consists of both biodegradable natural fiber as reinforcement and biodegradable polymer as a matrix. Therefore, bio-sourced polymers such as poly (lactic acid) (PLA), cellulose esters, poly (butylene succinate) (PBS) etc. are commercially introduced and replace petroleum based polymeric matrices such as polypropylene (PP), polyethylene (PE) because of the increasing price of petroleum and increasing awareness against pernicious effects of petroleum [7, 8, 9, 11, 12, 13, 16, 17]. In this work, we used PLA as a matrix and jute as a reinforcement to produce a complete biodegradable biocomposite. PLA is fermented from agricultural raw materials such as sugar, corn, beet, and potato in lactic acid [8, 13, 16, 18, 19] and has excellent mechanical properties [5, 7, 9, 20, 21, 22, 23], better biocompatibility [5, 7, 23], easily processable [7, 23], transparency [20], and biodegradability [5, 23]. Despite the properties, some modifications to PLA are required due to its brittleness, poor heat resistance, and low toughness properties [5, 9, 17, 22, 23, 24].

The –OH groups present in jute cellulose make jute hydrophilic in nature when combined with hydrophobic matrices. This relationship makes poor interface of fibers and more moisture absorbency that degrades mechanical properties [11, 12, 13, 16, 17, 20, 21, 22]. To develop the quality of composites, natural fibers undergo some chemical modifications including alkali treatment [2, 7, 22, 25, 26], bleaching [5, 22, 25, 27], silane treatment [5, 26, 28] and grafting [2, 5, 25]. These treatments strengthen fiber/matrix compatibility and hydrophobicity of fibers by lessening moisture uptake and accordingly ameliorate mechanical characteristics [29, 30, 31]. However, these treatments could not serve environmental purposes due to its corrosiveness, costliness and toxicity [19]. These very reasons triggered the researchers toward a new, eco-friendly, reliable and above all, inexpensive modification method.

A few physical pretreatments have been considered; steam explosion is one of them. This process removes hemicellulose, but due to upraised pressure, the lignin structure is altered [31, 32]. Therefore, the method is undependable with respect to the solubilization effect. Superheated steam (SHS) becomes a favorable substitute for steam explosion for lignocellulosic fibers, since SHS is performed at atmospheric pressure [29, 31]. SHS is a kind of unsaturated steam generated by applying an elevated boiling temperature with substantial pressure in saturated steam. The process incorporates energy savings, is economical for bulk production, has moderate risk factors, and is compatible with the environment [5, 19, 32, 33, 34]. Lately, SHS has contributed to the production of biomass, bio absorbents, and activated charcoal [5, 19, 32, 34]. The process attributes hemicellulose removal to the expanding hydrophobic nature, which increases the content of cellulose and lignin [5, 19]. Hence, superheated steam is welcomed for fabrication of biocomposites. In addition, it is a cost-effective, low-risk method for the surface modification of fibers. The superheated steam (SHS) process is applied to PALF [5], oil palm mesocarp fiber (OPMF) [19, 30, 33], bamboo fiber [35, 36], oil palm empty fruit bunches (OPEFB) [32, 37] and oil palm frond fiber (OPF) [38] for the fabrication of biocomposites.

Superheated steam treatment has been performed for modification on various fiber surfaces except jute fiber. Therefore, more research is required in this topic. In this experiment, the SHS modified jute fiber to enhance the interface bond between the fiber and the polymer matrix so that a compact, resilient, and biodegradable composite can be produced. Modification of the jute fiber surface by superheated steam treatment, production of cellulose fiber material, and improvement of the fiber-matrix interfacial bond are the main objective of this research work.

## 2 Experimental

### 2.1 Materials

**Figure 1:**
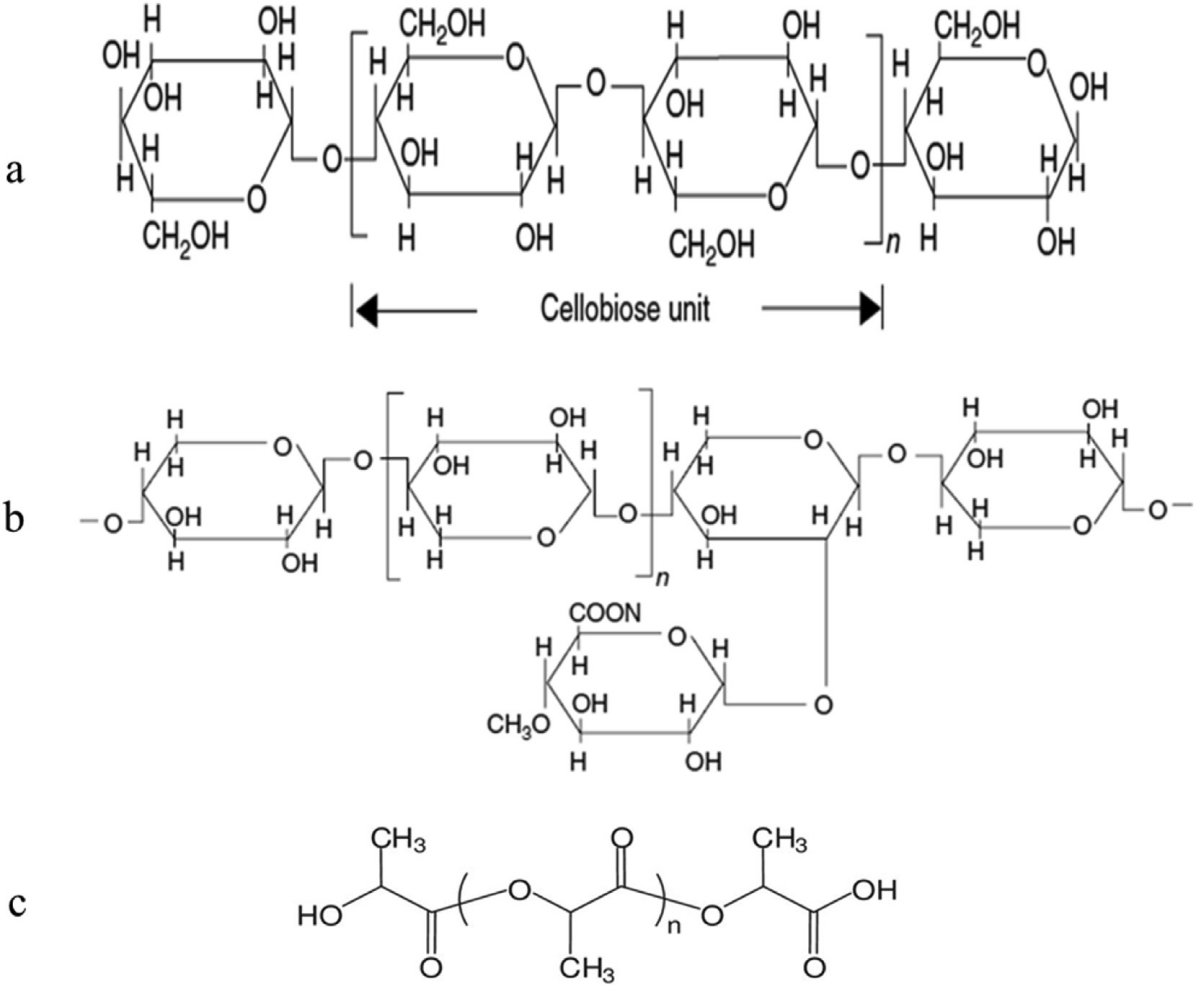
Chemical structures of alphacellulose of jute fiber (a), hemicellulose of jute fiber (b), and Poly (lactic acid) (PLA) (c) [39].

Bangladesh Jute Research Institute, Dhaka, Bangladesh supplied jute plant of variety C-145, Bangla White B (BWB) (Corchorus capsularis) in bundle form of long fibers. In exposure to sunlight, these samples were dried, then milled, filtered into 300–500 μm and preserved in airtight plastic bags for further investigation. The PLA polymer 3052D was supplied by Sigma Aldrich (Dhaka, Bangladesh) in a pellet form. It integrates a density of 1.252 g/cm^3^, a molecular mass of 93,500 g/mol and a boiling point between 150 and 160 °C. Figure 1 shows the chemical structures of alphacellulose and hemicellulose of jute fiber, and poly (lactic acid).

### 2.2 Alteration of jute by superheated steam treatment

**Figure 2:**
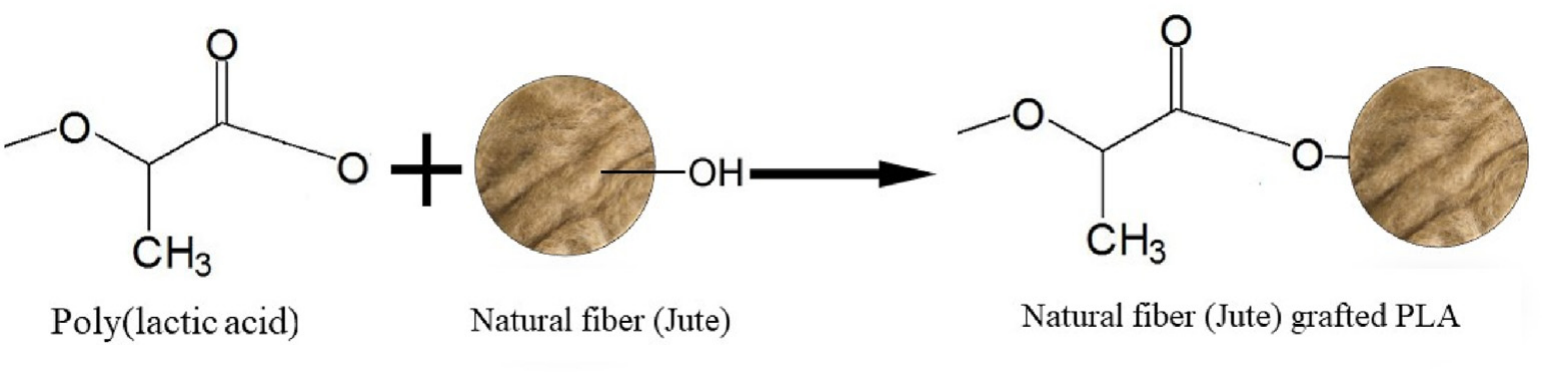
Hypothetical surface grafting reaction of jute with PLA chain by ring opening polymerization.

A superheated steam DC oven (DFA-5021, Naomoto Corporation, Japan) under standard pressure to modify the jute fiber by the SHS method as portrayed by Then et al. [30] Nordin et al. [29] and Challabi et al. [5]. The jute was dried in an SHS oven at 60 °C before the alteration process, then baked for 60 min in a SHS oven at temperatures of 170, 180, 190, 200, 210 and 220 °C. First, the SHS oven was made ready for jute modification and set in equilibrium condition. Subsequently, the jute was spread out on an aluminum foil plate and later placed in the SHS oven's heating chamber under specific conditions. After the treatment was completed, the fiber was removed from the chamber as soon as possible and cooled in a desiccator. The sample was then preserved in an airtight plastic bag. Jute shows supreme tensile properties for the fabrication of biocomposites when treated with 210 °C, compared to other treatments. Therefore, to determine the optimal treatment time, the sample was treated with various treatment times (30, 90, 120 min) following the same treatment process. Superheated steam (SHS) treated jute is denoted as SHS-Jute. Figure 2 indicates the hypothetical surface grafting reaction of jute with PLA chain by ring opening polymerization.

### 2.3 Biocomposites preparation

For the preparation of biocomposites, the jute samples were first dried. The oven dried jute was then mixed with PLA in a Brabender internal mixer to form biocomposites at 160 °C for 20 min as depicted by Challabi et al. [5]. Initially, the PLA pellets were dissolved for 2 min in the mixing chamber. The fibers were then included in the mixing chamber with PLA and the blending was carried out for 13 min through the compression molding process to generate layered materials having dimensions of 150 × 150 × 1 mm^3^ and 150 × 150 × 3 mm^3^ (length × width × thickness). Compression molding was carried out at a temperature of 160 °C and a pressure of 150 kg/cm^2^ for 5 min using a hydraulic hot press. Subsequently, the cooling was done at 30 °C for 5 min. The modified jute with PLA is denoted as SHS-Jute-PLA.

### 2.4 Characterizations

#### 2.4.1 FTIR study

FTIR recognized the functional units and chemical ingredients of raw jute and SHS-Jute using the IR Tracer-100, Shimadzu (Japan), equipped with the ATR configuration. The spectrums of the specimens were obtained extending 4000 cm^−1^ to 400 cm^−1^ wavenumbers.

#### 2.4.2 Morphological characterization

A scanning electron microscope (SEM), model Jeol-Jsm 7600, Japan, having an accelerating voltage of 10 kV was used to characterize the biocomposites.

#### 2.4.3 XRD study

An X-ray diffractometer (Rigaku Ultima IV, Japan) with a nickel-filtered Cu-Kα radiation at λ = 1.542 A (40 kV and 40 mA) was used to investigate the crystallinity of the bicomposites. The measurement of the samples was carried out in the range from 10° to 60° of 2θ at 4 min^−1^.

#### 2.4.4 Mechanical properties

The universal strength tester (Model Titan^5^, James Heal, UK), provided with a 5-kN load cell and a cross head speed of 5 mm min^−1^, examined the mechanical characteristics of the biocomposites at room temperature. Following the ASTM D638-5 (2000) testing instructions, five samples were examined. The tensile strength (TS), tensile modulus (TM), and elongation at break (EB) tests were calculated from the mean values and standard deviation.

The universal testing machine (Instron Corp., Norwood, MA, USA, Model 3365), provided with a 5-kN load cell, a cross head speed of 1.5 mm min^−1^ and a span length of 48 mm, was used to evaluate the bending characteristics (three-point testing) of the biocomposites. The test was conducted at room temperature on five samples having dimensions of 127.0 × 12.7 × 3.0 mm^3^. The procedure was adopted by ASTM D790 (2000). The mean values and standard deviation were shown by the bending strength (BS) and the bending modulus (BM).

An un-notched IZOD impact tester (Izod, Computerized, International Equipments, India), having 7.5-J pendulum, followed ASTM D256 (2000) instructions and recorded the biocomposites impact strength (IS) at room temperature on five specimens having measurements of 63.5 × 12.7 × 3.0 mm^3^ and then the mean values and standard deviations were determined.

#### 2.4.5 Swelling and water uptake profile analysis

According to EN 317 (1993) and ASTM D570, the thickness, swelling and water uptake of the biocomposites were assessed. For the water absorption test, test samples of 20 × 10 × 3 mm^3^ were cut from the bulk sample and kept at standard temperature. The primary thickness (T_0_) and weight (W_0_) of the dried samples were observed and recorded. After that, the test specimens were dipped in deionized water at room temperature for 24 h. The samples were then removed from the bath and properly wiped with tissue before their final thickness (T_24h_) and weight (W_24h_) were assessed. The water uptake % was evaluated using Eq. (1) and the thickness swelling was assessed using Eq. (2).

(1) $$\text{Water ​Uptake}\left(\text{\%}\right)=\frac{{\text{W}}_{24\text{h}}-{\text{W}}_{0}}{{\text{W}}_{0}}\times 100$$

(2) $$\text{Thickness ​Swelling}\left(\text{\%}\right)=\frac{{\text{T}}_{24\text{h}}-{\text{T}}_{0}}{{\text{T}}_{0}}\times 100$$

## 3 Results and discussion

### 3.1 Study of FTIR spectrums

**Figure 3:**
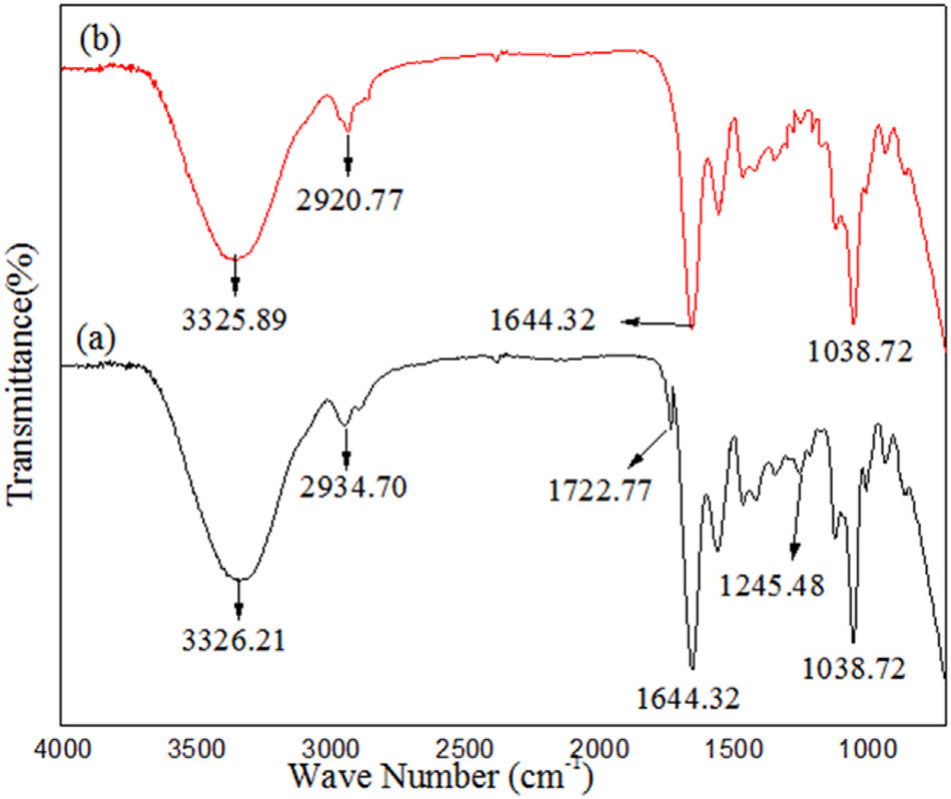
FTIR spectrums of (a) Untreated jute and (b) SHS-Jute.

FTIR spectra, chemical compositions and the peak positions of untreated jute and SHS-Jute are depicted in Figure 3 and Table 1, respectively. Figure 3 shows the FTIR spectrums of untreated jute and SHS-Jute at 210 °C for 60 min, under these conditions which have yielded biocomposites with optimum tensile properties. The typical cellulose absorption bands are shown in the pure jute spectrum (Figure 3(a)): –OH group at 3326.21 cm^−1^ and C–H group 2934.70 cm^−1^ respectively [40, 41]. A similar peak appeared at 3325.89 cm^−1^ and 2920.77 cm^−1^ in the spectrums (Figure 3(b)) of SHS-Jute which became weaker after oven-dry treatment. This is because the cellulose –OH groups were transformed to hydrogen bonds by oven-dry treatment of jute, which reduced the vibration of both C–H bonds and –OH groups. The peaks at 1722.77 cm^−1^, 1644.32 cm^−1^ and 1245.48 cm^−1^ were due to the C=O stretching of carboxylic acid in hemicellulose, –CH_2_ stretching of cellulose and the C–O stretching of lignin, respectively [42, 43, 44]. Furthermore, in the case of SHS-Jute, there was no peak at 1245.48 cm^−1^ and 1722.77 cm^−1^ which indicates the partial removal of hemicellulose and lignin, respectively. Sena Neto et al. also reported similar results [43]. The absence of these two bands in SHS-Jute suggests that residual components were effectively removed during oven-drying of the jute with an optimum treatment time and temperature.

**Table 1:** Assignments and peak positions in FTIR spectrums of untreated jute and SHS-Jute.

Stretching	Untreated jute (cm^−1^)	Superheated Steam Jute (SHS-Jute) (cm^−1^)	References
–OH stretching of cellulose	3326.21	3325.89	[45]
C–H stretching of cellulose	2934.70	2920.77	[37]
–CH_2_ stretching of cellulose	1644.32	1644.32	[44]
C=O stretching of hemi-cellulose	1722.77	–	[41]
C–O stretching of lignin	1245.48	–	[42]
C–OH stretching	1038.72	1038.72	[46]

### 3.2 SEM analysis

**Figure 4:**
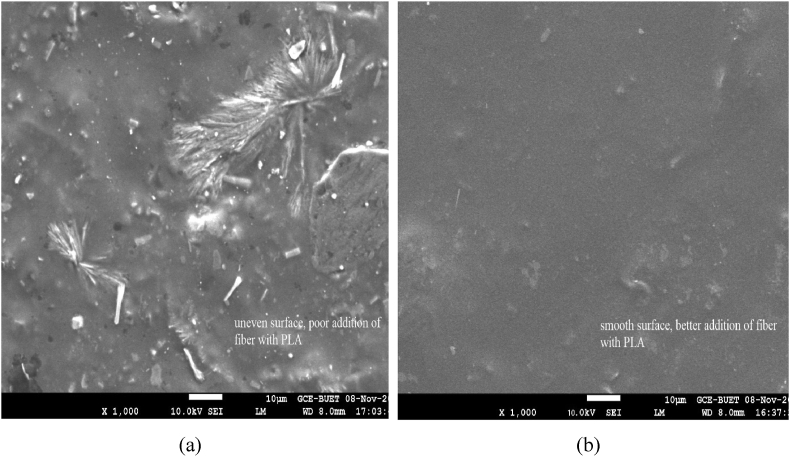
SEM image of (a) Untreated Jute-PLA (b) SHS-Jute-PLA biocomposites.

Figure 4 shows the morphology of the surface of the biocomposites. The uneven surfaces with no holes or gaps of the SHS-Jute-PLA biocomposites (Figure 4(b)) may be due to removing hemicellulose and other impurities from the surface. The SHS treatment improves fiber properties removing holes, gaps from surface and makes the fiber surface rougher. The rough surface enhances fiber-polymer bonding. The SEM image of the Jute-PLA biocomposite (Figure 4(a)) showed gaps, holes and cracks which could have contributed to their inferior material properties like mechanical and dimensional, resulting from a lower interface adhesion due to high hemicellulose content for the untreated jute and the brittle behavior of the hydrophobic PLA [45]. This could explain that the improved characteristics of the SHS-Jute-PLA biocomposite and the formation of a nice structure on the surface with 210 °C treatment for a time of 60 min.

### 3.3 XRD evaluation

**Figure 5:**
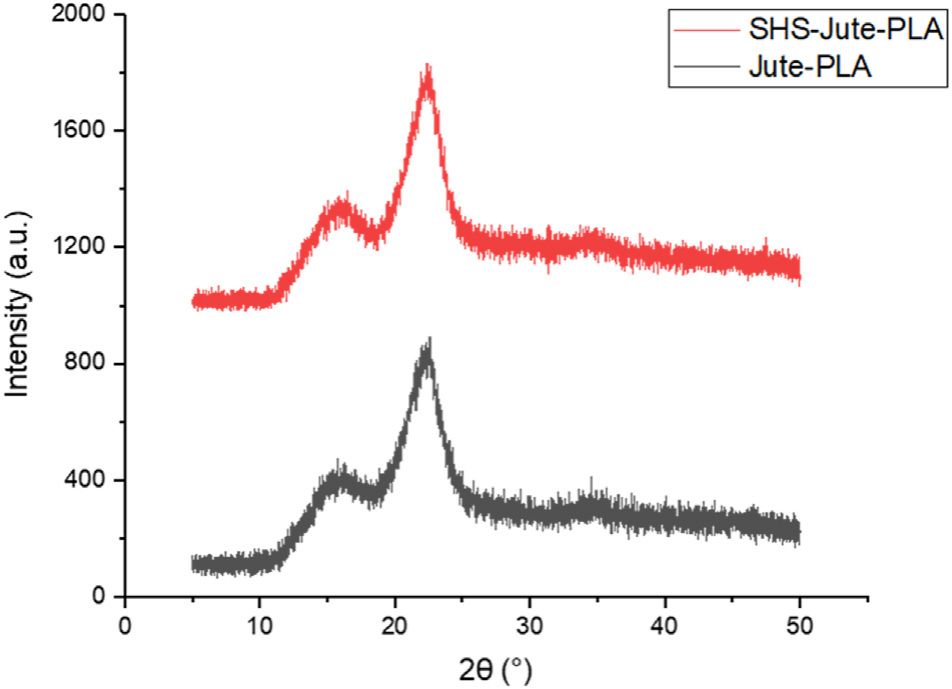
X-ray diffraction images of Jute-PLA and SHS-Jute-PLA.

The X-ray diffraction technique was employed to evaluate the crystalline structure of the biocomposites. From the Figure 5, The Jute-PLA and SHS-Jute-PLA biocomposites showed two distinctive XRD patterns at almost 2θ ≈ 16.8° and 2θ ≈ 22°; these peaks are aligned with the unique reflections of the cellulose 1 crystalline polymorph, related observations reported by Sena Neto [43] and Jalil et al. [47]. The existence of these two distinctive peaks can be ascribed to the presence of jute and PLA in the composite. Moreover, the XRD images of the SHS-Jute-PLA were alike Jute-PLA composite, showing that Superheated steam process did not alter cellulose’s crystalline structure [22]. Furthermore, the strength of the diffraction peak increased, indicating that the crystallinity of the SHS-Jute-PLA biocomposite increased [25]. From the results, the crystallinity index of SHS-Jute-PLA and Jute-PLA is 68.69% and 52.23%, respectively. SHS treatment removes hemicellulose and impurities which improves the crystallinity index. This outcome claims the enrichment in the dimensional stability of the SHS-Jute-PLA biocomposite. Higher crystallinity of biocomposites is regarded as the key elements for enhanced dimensional stability, according to Mathew et al. [48]. As a result, it may be concluded that the crystallinity of biocomposites was preserved during processing.

### 3.4 Tensile properties

The excellent efficacy of super-heated steam (SHS) treatment on Jute-PLA biocomposite was constructively evaluated and the recorded results were linked to untreated Jute-PLA biocomposite. The findings of different tensile properties of Jute-PLA and SHS-Jute-PLA biocomposites are assembled in Table 2.

**Table 2:** Tensile performance of Jute-PLA and SHS-Jute-PLA samples.

Samples	Temperature (°C)	Time (minute)	Tensile Strength (TS), MPa	Elongation at Break (EB), %	Tensile Modulus (TM), GPa
PLA	–	–	64.55 ± 0.19	6.60 ± 0.32	1.13 ± 0.04
Jute-PLA	–	–	48.81 ± 1.76	3.65 ± 0.15	1.40 ± 0.03
SHS-Jute-PLA	170	60	48.68 ± 1.12	3.59 ± 0.01	1.59 ± 0.06
	180	60	48.69 ± 1.14	3.82 ± 0.19	1.60 ± 0.08
	190	60	52.24 ± 1.42	3.75 ± 0.21	1.61 ± 0.07
	200	60	52.01 ± 1.14	3.84 ± 0.17	1.58 ± 0.04
	210	30	55.01 ± 1.34	3.74 ± 0.17	1.61 ± 0.06
		60	59.94 ± 1.52	4.28 ± 0.15	1.63 ± 0.04
		90	54.10 ± 1.61	3.83 ± 0.02	1.63 ± 0.05
		120	53.43 ± 2.16	3.57 ± 0.16	1.81 ± 0.10
	220	60	43.20 ± 1.33	3.01 ± 0.17	1.56 ± 0.02

The tensile strength of the SHS-Jute-PLA biocomposite was endured approximately identical as that of the Jute-PLA composite up to the temperature of 180 °C at 60 min, while the elongation at break and tensile modulus increased (See in Table 2). SHS-Jute-PLA biocomposites’ TS and TM increased as treatment temperature climbed from 190 to 210 °C but decreased at 220 °C. It is evident from the Table 2 that the tensile properties were found in decreasing manner after 210 °C. The better results were found for the sample of SHS-Jute-PLA biocomposites at 210 °C for 60 min and this time was selected for the next other study.

The TS of the SHS-Jute-PLA biocomposites was increased with increasing treatment time up to 60 min and then decreased. However, TM continued to be constant from 60 to 90 min, peaking at 120 min.

The various impurities including hemicellulose were removed from the fiber surface through SHS treatment. Consequently, the adhesive properties of the SHS-Jute-PLA biocomposite were improved. Because SHS was employed to increase the surface adherence of PLA and jute. The interfacial bonding and mechanical interlocking between the fiber and polymer matrix were improved. Moreover, the hydrophobicity of SHS-Jute increased due to decreased water absorption [29]. Thus, the tensile characteristics of the SHS-Jute-PLA biocomposite increased.

The removal of hemicellulose increases the tensile strength, but the substantial amounts of acetic acid could be released at high temperature and longer treatment time. This acid may boost up the cellulose degradation and consequently, the tensile properties of biocomposite materials decrease [30].

### 3.5 Bending and impact properties

Because of the hydrophilicity of jute and hydrophobicity of PLA, the interfacial bond between jute and PLA becomes weak. As a result, the bending properties of the Jute-PLA biocomposites differ before and after SHS modification on the jute fiber. Table 3 shows the outcomes of the bending and impact analysis performed on the Jute-PLA and SHS-Jute-PLA biocomposites at 210 °C for 60 min. From the table it is obvious that bending strength of SHS-Jute-PLA is higher than that of Jute-PLA and it is approximately 16%. This is because of the interfacial bond between jute and PLA, resulted due to increased interfacial adhesion between jute and PLA. The bending modulus of SHS-Jute-PLA is increased approximately 8% than Jute-PLA. This inequality may be due to the short lengths of the jute fiber, which also may be dispersed unevenly in the biocomposites. The impact strength of SHS-Jute-PLA is noted approximately 158.70 that is about 16.70% more than Jute-PLA. The SHS-Jute-PLA imply a higher increase due to the strong bond between jute and PLA resulting from the modification of SHS on the jute fiber. The biocomposite can retain more impact energy without cracking.

**Table 3:** Bending and impact characteristics of Jute-PLA and SHS-Jute-PLA biocomposites.

Biocomposite	Bending Strength, (MPa)	Bending Modulus, (GPa)	Impact Strength, (J/m)
Jute-PLA	72.26 ± 1.54	5.50 ± 0.20	135.75 ± 3.08
SHS-Jute-PLA	83.56 ± 0.48	5.95 ± 0.18	158.70 ± 6.54

### 3.6 Swelling and Water Uptake Profile

**Figure 6:**
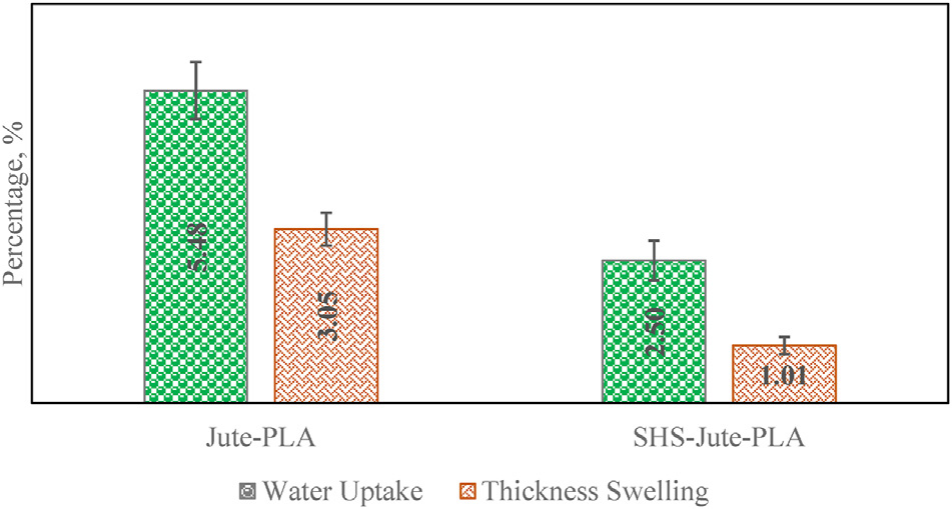
Swelling and Water Uptake Profile of SHS-Jute-PLA and Jute-PLA biocomposites.

Water absorption is one of the main disadvantages of natural fiber composites due to the presence of a hydrophilic character. The applications of composites are limited due to the propensity of natural fibers to regain moisture and swell when encountered water and moist environment [49]. Water uptake and thickness swelling of SHS-Jute-PLA and Jute-PLA after 24 h in water at 210 °C for 60 min shown in Figure 6. Both biocomposites have increased thickness swelling and water uptake after 24 h of immersion in water, due to the existence of hydroxyl groups in fiber, which allow hydrogen bonding with water molecules and increase water absorption [49]. However, when compared to untreated Jute-PLA biocomposite, SHS-Jute-PLA biocomposite has a lower rate of thickness swelling and water absorption.

Figure 6 shows that as compared to Jute-PLA biocomposite, the water uptake of SHS-Jute-PLA (2.5%) is reduced by 54%. This can be explained by the decrease in hemicellulose caused by SHS treatment, the increment in cellulose content in the fiber, and the decrease in free space caused by the improved binding force. The thickness swelling of SHS-Jute-PLA and Jute-PLA biocomposites is 3.05% and 1.01%, respectively. This result exhibits the thickness swelling of the SHS-Jute-PLA biocomposite has been reduced by 48%. This is attributable to the SHS-Jute-PLA biocomposite's low water uptake, as thickness swelling is proportional to the amount of water absorbed. The summary of the strength of various Jute-PLA composites prepared by various surface treatment method is shown in Table 4.

**Table 4:** Summary of the strength of various Jute-PLA composites prepared by various surface treatment method.

Composites	Surface treatment process	Tensile strength (MPa)	Bending strength (MPa)	Impact strength (KJ/m^2^)	Reference
Jute-PLA	Superheated steam treatment	59.94 ± 1.52	83.56 ± 0.48	0.16 ± 0.006	This work
Jute-PLA	Alkali treatment	65	112	5.3	[21]
Jute-PLA	Chemical treatment	45.67	57	0.037	[50]
Jute-PLA	Alkali treatment	55	110	1.6	[51]
Jute-PLA	–	50	78	4.8	[52]

## 4 Conclusion

In this existing work, the SHS process can be used as an alternative of chemical finishing of jute fibers and can contribute much to build a chemical free environment. SEM analysis confirmed that fiber surface smoothness has increased due to removing hemicellulose and impurities by the SHS process. This makes the jute samples less hydrophilic in nature which is suitable for biocomposites preparation. The tensile properties test showed that the tensile strength, tensile modulus and elongation at break have been increased by approximately 23%, 29% and 17% respectively of SHS-Jute-PLA. The bending and impact properties showed that the bending strength, bending modulus, and impact strength have increased by approximately 15.64%, 8%, and 16.7%, respectively. FTIR spectroscopy analysis showed that PLA has formed a strong interaction with SHS-Jute fibers and this analysis presented a good compatibility with the SEM results. The increase in crystallinity of the biocomposites was also observed from the XRD test. The water uptake% and thickness swelling of the SHS-Jute-PLA biocomposite system were less pronounce. Therefore, it can be said that SHS is an effective, economic, compatible and eco-friendly method that can be able to improve the dimensional and physico-mechanical properties of the biocomposites. These improved properties will help the biocomposites commercialization. It can be concluded from this work that the SHS treatment can be used as an alternative to chemical methods for modifying the jute surface for biocomposites manufacturing.

## Declarations

### Author contribution statement

Md. Abdul Alim: Conceived and designed the experiments; Performed the experiments; Wrote the paper.

Md. Moniruzzaman: Conceived and designed the experiments; Analyzed and interpreted the data; Wrote the paper.

Md. Muzaher Hossain, Wahiduzzaman: Performed the experiments; Analyzed and interpreted the data; Contributed reagents, materials, analysis tools or data.

Md. Reazuddin Repon: Conceived and designed the experiments; Performed the experiments; Analyzed and interpreted the data; Wrote the paper.

Ismail Hossain: Conceived and designed the experiments; Analyzed and interpreted the data; Contributed reagents, materials, analysis tools or data.

Mohammad Abdul Jalil: Conceived and designed the experiments; Performed the experiments; Contributed reagents, materials, analysis tools or data; Wrote the paper.

### Funding statement

This research did not receive any specific grant from funding agencies in the public, commercial, or not-for-profit sectors.

### Data availability statement

Data included in article/supplementary material/referenced in article.

### Declaration of interests statement.

The authors declare no conflict of interest.

### Additional information

No additional information is available for this paper.
